# The role of ethnicity and native-country income in multiple sclerosis: the Italian multicentre study (MS-MigIT)

**DOI:** 10.1007/s00415-024-12214-6

**Published:** 2024-02-16

**Authors:** Alessia Bianchi, Domenica Matranga, Francesco Patti, Laura Maniscalco, Silvy Pilotto, Massimiliano Di Filippo, Mauro Zaffaroni, Pietro Annovazzi, Antonio Bertolotto, Claudio Gasperini, Esmeralda Quartuccio, Diego Centonze, Roberta Fantozzi, Alberto Gajofatto, Francesca Gobbin, Doriana Landi, Franco Granella, Maria Buccafusca, Girolama Alessandra Marfia, Clara Chisari, Paola Naldi, Roberto Bergamaschi, Giacomo Greco, Ignazio Roberto Zarbo, Vincenzo Rizzo, Monica Ulivelli, Daiana Bezzini, Lucia Florio, Michelangelo Turazzini, Maria Di Gregorio, Maura Pugliatti, Giuseppe Salemi, Paolo Ragonese, the the MS-MigIT Study Group

**Affiliations:** 1 Department of Biomedicine, Neurosciences and Advanced Diagnostic, University of Palermo, Via Gaetano La Loggia 1, 90129 Palermo, Italy; 2 Department of Neuroinflammation, Queen Square Multiple Sclerosis Centre, University College London, London, UK; 3 Interdepartmental Research Centre On Migration (CIR “Migrare”), University of Palermo, Palermo, Italy; 4 Department of Health Promotion, Mother and Child Care, Internal Medicine and Medical Specialties, University of Palermo, Palermo, Italy; 5 Department of Medical and Surgical Sciences and Advanced Technologies, University of Catania, Catania, Italy; 6 Department of Neuroscience and Rehabilitation, University of Ferrara, Ferrara, Italy; 7 Section of Neurology, Department of Medicine, University of Perugia, Perugia, Italy; 8 Multiple Sclerosis Centre, Hospital of Gallarate, ASST Della Valle Olona, Gallarate, Italy; 9 Ospedale Koelliker, Turin and Neuroscience Institute Cavalieri Ottolenghi, Orbassano, Italy; 10 Department of Neurology, San Camillo-Forlanini Hospital, Rome, Italy; 11 Unit of Neurology, Department of Neurorehabilitation, Istituto di Ricovero e Cura a Carattere Scientifico (IRCCS) Neuromed, Pozzilli, Italy; 12 Department of Systems Medicine, Tor Vergata University, Rome, Italy; 13 Department of Neuroscience, Biomedicine and Movement Sciences, University of Verona, Verona, Italy; 14 Multiple Sclerosis Clinical and Research Unit, Tor Vergata University Hospital, Rome, Italy; 15 Department of Medicine and Surgery, University of Parma, Parma, Italy; 16 Department of Clinical and Experimental Medicine, Unit of Neurology and Neuromuscular Diseases, University of Messina, Messina, Italy; 17 Department of Translational Medicine, University of Piemonte Orientale, Novara, Italy; 18 IRCCS Mondino Foundation, Pavia, Italy; 19 Department of Medicine, Surgery and Pharmacy, University of Sassari, Sassari, Italy; 20 Department of Medicine, Surgery and Neuroscience, University of Siena, Siena, Italy; 21 IRCCS Casa Sollievo Della Sofferenza, San Giovanni Rotondo, Italy; 22 Stroke Unit, Ospedale Mater Salutis, Legnago, Italy; 23 Azienda Ospedaliera Universitaria OO.RR. S.Giovanni di Dio e Ruggi d’Aragona, Salerno, Italy

**Keywords:** Multiple sclerosis, Migration, Ethnic group, Geographical factors, Income, Case–control study

## Abstract

**Objective:**

Multiple sclerosis (MS) is a complex disorder in which environmental and genetic factors interact modifying disease risk and course. This multicentre, case–control study involving 18 Italian MS Centres investigated MS course by ethnicity and native-country economic status in foreign-born patients living in Italy.

**Methods:**

We identified 457 MS patients who migrated to Italy and 893 age- and sex-matched native-born Italian patients. In our population, 1225 (93.2%) subjects were White Europeans and White Northern Americans (WENA) and 89 (6.8%) patients were from other ethnical groups (OEG); 1109 (82.1%) patients were born in a high-income (HI) Country and 241 (17.9%) in a low-middle-income (LMI) Country. Medical records and patients interviews were used to collect demographic and disease data.

**Results:**

We included 1350 individuals (973 women and 377 men); mean (SD) age was 45.0 (11.7) years. At onset, 25.45% OEG patients vs 12.47% WENA (p = 0.039) had > 3 STIR spine lesions. At recruitment, the same group featured mean (SD) EDSS score of 2.85 (2.23) vs 2.64 (2.28) (p = 0.044) reached in 8.9 (9.0) vs 12.0 (9.0) years (p = 0.018) and underwent 1.10 (4.44) vs. 0.99 (0.40) annual MRI examinations (p = 0.035). At disease onset, patients from LMI countries had higher EDSS score than HI patients (2.40 (1.43) vs 1.99 (1.17); p = 0.032).

**Discussion:**

Our results suggested that both ethnicity and socio-economic status of native country shape MS presentation and course and should be considered for an appropriate management of patients. To the best of our knowledge, this is the first study reporting on the impact of ethnicity in MS at an individual level and beyond an ecological population-perspective.

**Supplementary Information:**

The online version contains supplementary material available at 10.1007/s00415-024-12214-6.

## Introduction

Multiple sclerosis (MS) is a chronic, immune mediated inflammatory and degenerative disorder of the central nervous system (CNS). Epidemiological evidence indicates that both genetic and environmental factors are involved in disease development and course [1] through interaction [1–3].

In 2020 the Multiple Sclerosis International Federation (MSIF) reported a remarkable variation in the disease prevalence and incidence across different geographical areas [4]. *Ethnicity* is a complex concept that has become a topic of great interest over the last decades. The term refers to a cultural identity, often based on shared culture, religion, traditions, and ancestry, and therefore involving both environmental and genetic factors [5, 6]. Ethnicity is therefore a social construct that may be useful as a lens through which evaluate disparities in health care [7]. Indeed, several studies suggested that ethnicity could play a role in determining the geographical differences observed in MS [8]. The economic status of a given Country has also been reported in association with geographical variations of MS frequency. Indeed, not only the income of a Country influences the population’s lifestyle and their exposure to specific environmental factors, but it could also determine a delay in the diagnosis or restrict the access to disease-modifying therapies (DMTs) [9, 10].

The overarching aim of our study was to investigate whether and how exposures from Country of origin could influence MS characteristics at onset and disease course. To achieve this target, we defined our aims as follows: (1) to compare MS clinical and radiological features between ‘foreign-born patients’ and patients born in Italy, (2) to compare MS clinical and radiological features between patients from different ethnic groups, and (3) to compare MS clinical and radiological features between patients born in low- and middle-income (LMI) Countries versus patients born in high-income (HI) Countries.

## Method and participants

Eighteen MS Centres in Italian Public Hospitals participated in this multicentre, case–control study. Data were collected between January 2018 and December 2020. We identified 457 patients who were born outside Italy (*foreign-born patients*), had a confirmed diagnosis of MS according to revised McDonald criteria [11, 12], and had attended an Italian MS Centre. For each foreign-born patient, we recruited two age- (± 6 months) and sex-matched native-born Italian patients, and a total of 893 native-born Italian MS patients were enrolled. Proceeding from the results obtained in a pilot, single-centre study to compare foreign-born patients versus native-born Italian patients, we calculated that a population of 800 MS patients (foreign-born:Italian = 1:2) would be necessary to detect a difference of 1.0 point in EDSS score and a difference of 1.0 point in EDSS change over time between the groups at 0.8 power and 5% significance level.

MS patients were categorised by ethnicity and gross national income (GNI) per capita of their native Country. Ethnicities were obtained from medical records as self-reported by patients at the first visit at MS Centre or obtained directly from patients. As most of clinical trials and research studies are conducted in North America and Europe and included White people, we compared two macro-groups: White Europeans and White North Americans (WENA), who are traditionally well-represented in clinical trials and research studies, versus other ethnical groups (OEG), which includes all the other underrepresented groups [13, 14].

Countries were assigned to a specific income group according to the 2018 World Bank Atlas [15]: (1) low- and middle-income (LMI) economies are defined as those with a GNI per capita of less than United States (US) $12,056, while high-income (HI) economies are those with a GNI per capita of US $12,056 or higher.

Medical records were used to collect data on disease features at onset, diagnosis, and at recruitment time. We also obtained demographic information, including age, sex, native-Country of parents, and age at migration to Italy.

This study was conducted according to the Helsinki Declaration. The study protocol was approved by the local institutional review board of the University Hospital “Policlinico Paolo Giaccone” in Palermo (approval number: 10/2018). All patients gave informed consent upon admission to the study.

### Statistical analysis

Patients were classified according to their native Country (native-born Italian patients vs foreign-born patients), ethnicity (WENA vs OEGs), and income of the native Country (HI Countries vs LMI Countries). Data were analysed using Stata IC/15.1 (StataCorp LLC, Texas, TX, USA) software, and a p < 0.05 was chosen as the statistical significance cut-off.

Descriptives were reported with counts and percentages for categorical variables, and means ± standard deviations (SD) for continuous variables. Median and interquartile range (IQR) were used when the variable distribution was not normally distributed.

The association with the response was assessed through one-way ANOVA or the equality of k-medians test, in case of skewed distributions. For the scope of multivariate analysis, quantitative explanatory variables were categorized using the median as cut-off (EDSS at onset as 0–2.5, 3–5, > 5; EDSS at follow-up as 0–3.5, 4–6 and > 6).

Due to the multicentric study design and considering the binary nature of the response variables, we used two-level variance component logistic regression models with a hierarchical structure given by patients nested within Centres. By incorporating random effects, we could address possible biases associated to the heterogeneity in the clinical approach. Variables to be included in these models were those statistically significant at univariable analysis. Results were expressed as adjusted odds ratio (ORs) and 95% confidence interval (CIs) for fixed effects. The estimated variance among centres with 95% CI was given to assess heterogeneity.

To assess robustness in the presence of missing data, the analysis was replicated on multiple imputed datasets using the STATA module*-mi* impute chained. This procedure accommodates arbitrary missing-value patterns, with missing values imputed iteratively across multiple variables using chained equations—a sequence of univariate imputation methods with fully conditional specification (FCS) of prediction equations. Subsequently, the STATA command *mi estimate, cmdok: melogit* was employed to estimate a two-level variance components model on multiple imputed datasets.

## Results

Overall, 1350 MS patients were enrolled in the study, counting for 457 foreign-born patients and 893 patients born in Italy. The population included 973 (72.1%) women and 377 (27.9%) men (woman to man ratio = 2.58) and the mean (SD) age at recruitment was 45.0 (11.7) years (Table 1). In this population, 1225 (93.2%) subjects were WENA, of whom 333 (27.2%) born in a foreign Country, while 89 (6.8%) were OEG, 88 (98.9%) of whom born abroad. We found 18 (1.4%) Black Africans, 39 (3.0%) Middle Eastern and North African Arabs, 2 (0.2%) Eastern Asians, 1 (0.1%) Creole Caribbeans, 23 (1.8%) South American Hispanics, and 6 (0.5%) Middle Eastern and North African Jewish [7]. Ethnicity was not available for 36 (2.7%) patients. Considering the income, we found that 1109 (82.1%) patients were born in a HI Economy and 241 (17.9%) in a LMI Economy: of the 89 OEG patients, 78 (87.6%) were born in a LMI Country, while of the 1225 WENA, 159 (13.0%) were in the LMI Country group.

**Table 1:** Comparison between native-born Italian patients and foreign-born patients

	Native-born Italian patients (n = 893)	Foreign-born patients (n = 457)	p value
Demographic data			
Age, mean ± sd	45.0 ± 11.8	45.0 ± 11.6	0.958
Female:male (ratio)	642:251 (2.56)	331:126 (2.63)	0.835
Familiarity for AI disease, prevalence (%)	115/824 (13.96%)	74/376 (19.68%)	0.012
Comorbidity, prevalence (%)	438/883 (49.60%)	192/377 (50.93%)	0.667
Psychiatric comorbidity, prevalence (%)	70/882 (7.94%)	33/395 (8.35%)	0.800
Other CNS disease, prevalence (%)	24/881 (2.72%)	15/395 (3.80%)	0.303
Onset and diagnosis data			
Age at onset, mean ± sd	30.1 ± 10.1	30.1 ± 10.1	0.989
Time-gap from onset to diagnosis gap (months)^, mean ± sd	32.3 ± 57.4	37.6 ± 63.9	0.690
EDSS (score)^			
Median (range)	2.0 (0.0–7.0)	2.0 (0.0–8.0)	0.016
Mean ± sd	1.96 ± 1.16	2.26 ± 1.33	
Type of onset^, prevalence (%)			
Supratentorial	240/892 (26.91%)	125/447 (27.96%)	0.682
Optic pathway	237/892 (26.57%)	109/448 (24.33%)	0.377
Brainstem	211/892 (23.65%)	118/447 (26.40%)	0.271
Cerebellar	102/892 (11.43%)	66/447 (14.77%)	0.083
Spinal cord	273/892 (30.61%)	152/447 (34.00%)	0.208
Polysymptomatic	193/892 (21.64%)	99/451 (21.95%)	0.895
Progression at onset^, prevalence (%)	136/868 (15.67%)	88/433 (20.32%)	0.036
Brain MRI: number of T2w/FLAIR lesions^, prevalence (%)			
0 lesions	14/676 (2.07%)	5/311 (1.61%)	0.405
1–3 lesions	99/676 (14.64%)	40/311 (12.86%)	
4–10 lesions	260/676 (38.46%)	137/311 (44.05%)	
≥ 10 lesions	303/676 (44.82%)	129/311 (41.48%)	
Brain MRI: distribution of T2w/FLAIR lesions^, prevalence (%)			
Periventricular	541/597 (90.62%)	253/281 (90.04%)	0.784
Juxtacortical	390/581 (67.13%)	188/275 (68.36%)	0.718
Infratentorial	335/593 (56.49%)	142/274 (51.82%)	0.199
Corpus callosum	221/588 (37.59%)	104/272 (38.24%)	0.855
Brain MRI: atypical of lesions^, prevalence (%)	14/574 (2.44%)	10/323 (3.10%)	0.558
Brain MRI: number of T1w lesions^, prevalence (%)			
0 lesions	297/625 (47.52%)	118/290 (40.69%)	0.128
1–3 lesions	161/625 (25.76%)	82/290 (28.28%)	
4–10 lesions	121/625 (19.36%)	58/290 (20.00%)	
≥ 10 lesions	46/625 (7.36%)	32/290 (11.03%)	
Brain MRI: contrast lesions^, mean ± sd	0.83 ± 2.44	0.72 ± 1.52	0.802
Brain MRI: persistent contrast lesions^, prevalence (%)	28/677 (4.14%)	16/306 (5.23%)	0.443
Spine MRI: number of STIR lesions^, prevalence (%)			0.200
0 lesions	216/628 (34.39%)	103/297 (34.68%)	
1–3 lesions	336/628 (53.50%)	146/297 (49.16%)	
4–10 lesions	72/628 (11.46%)	46/297 (15.49%)	
≥ 10 lesions^§^	4/628 (0.64%)	2/297 (0.67%)	
Spine MRI: atypical of lesions^, prevalence (%)	5/306 (1.63%)	3/184 (1.63%)	0.998
Spine MRI: contrast lesions^, mean ± sd	0.24 ± 0.52	0.25 ± 0.67	0.509
MRI: Barkhof criteria^, prevalence (%)	581/716 (81.15%)	264/353 (74.79%)	0.016
Abnormal evoked potentials^, prevalence (%)			
VEPs	257/460 (55.87%)	112/211 (53.08%)	0.500
BAEPs	79/285 (27.72%)	27/111 (24.32%)	0.493
MEPs	79/235 (33.62%)	41/110 (37.27%)	0.506
SEPs	171/335 (51.04%)	71/159 (44.65%)	0.184
Positive OCBs^, prevalence (%)	484/593 (81.62%)	223/268 (83.21%)	0.573
Recruitment data			
Disease duration (years)^, mean ± sd	12.1 ± 9.0	10.8 ± 8.9	0.013
EDSS (score)°			
Median (range)	1.5 (0.0–9.5)	2.0 (0.0–9.0)	0.009
Mean ± sd	2.59 ± 2.30	2.77 ± 2.21	
EDSS changes (point in score)°, median (range)	0.0 (− 3.0 to 6.5)	0.0 (− 4.0 to 5.5)	0.896
Time-gap from onset to EDSS 4.0 (years)°, mean ± sd	6.8 ± 7.9	6.4 ± 7.2	0.625
Time-gap from onset to EDSS 6.0 (years)°, mean ± sd	9.2 ± 8.6	8.9 ± 9.0	0.837
Relapses in the first 3 years within onset°, mean ± sd	1.94 ± 1.56	1.82 ± 1.46	0.154
Annual relapse rate°, mean ± sd	0.79 ± 1.27	0.53 ± 0.54	0.522
Annual clinical visit rate°, mean ± sd	2.30 ± 1.71	2.19 ± 1.72	0.284
Annual MRI scan rate°, mean ± sd	0.99 ± 0.40	1.01 ± 0.42	0.482
Progression at follow-up°, prevalence (%)	198/889 (22.27%)	95/452 (21.02%)	0.599
Time on first DMT (years)°, mean ± sd	3.8 ± 4.6	4.2 ± 4.9	0.112
Number of DMTs°, mean ± sd	2.00 ± 1.24	1.72 ± 1.17	< 0.001
Therapeutic approach°, frequency (%)			0.222
Induction	159/808 (19.68%)	66/394 (16.75%)	
Escalation	649/808 (80.32%)	328/394 (83.25%)	

**^**Analysis adjusted for age and sex

°Analysis adjusted for age and disease duration

^**§**^Adjacent categories with frequency < 5 were collapsed for p value calculation

A comparison of the main demographic and clinical characteristics between foreign-born patients and native-born Italian patients is reported in Table 1. We found that foreign-born patients had higher prevalence of family history for autoimmune (AI) diseases when compared to native-born Italian patients (p = 0.036). At onset, the former group also reported higher prevalence of progressive phenotypes (p = 0.036) and higher mean Expanded Disability Status Scale (EDSS) score (p = 0.016). At recruitment, native-born Italian patients had longer disease duration (p = 0.013), but lower mean EDSS score (p = 0.009). However, this significance was lost after adjusting for EDSS score at onset (p = 0.357). Finally, native-born Italian patients had underwent more disease-modifying treatments (DMTs) than foreign-born patients (p < 0.001).

Clinical, paraclinical, and radiological characteristics of WENA and OEG at onset, baseline, and recruitment are detailed in Table 2. At the time of diagnosis, 55/62 (88.7%) OEG had > 3 T2-weighted (T2w) lesions at the brain magnetic resonance imaging (MRI) scan compared with 748/896 (83.5%) WENA (p = 0.025), while > 3 Short-TI Inversion Recovery (STIR) lesions in the spinal cord were detected in 14/55 (25.5%) of OEG versus 105/842 (12.5%) WENA patients (p = 0.006).

**Table 2:** Comparison between White Europeans and North Americans (WENA) patients and other ethnical group (OEG) patients

	WENA patients (n = 1225)	OEG patients (n = 89)	p value
Demographic data			
Age, mean ± sd	45.2 ± 11.7	43.4 ± 12.4	0.159
Female:male (ratio)	884:341 (2.59)	63:26 (2.42)	0.780
Familiarity for AI disease, prevalence (%)	168/1105 (15.20%)	14/73 (19.18%)	0.363
Comorbidity, prevalence (%)	575/1150 (50.00%)	41/79 (51.90%)	0.744
Psychiatric comorbidity, prevalence (%)	97/1167 (8.31%)	6/79 (7.59%)	0.823
Other CNS disease, prevalence (%)	36/1166 (3.09%)	3/79 (3.80%)	0.726
Onset and diagnosis data			
Age at onset, mean ± sd	30.0 ± 10.1	31.1 ± 10.6	0.325
Time-gap from onset to diagnosis gap (months), mean ± sd	33.9 ± 58.1	33.4 ± 76.5	0.939
EDSS (score)^			
Median (range)	2.0 (0.0–8.0)	2.0 (0.0–7.0)	0.132
Mean ± sd	2.04 ± 1.19	2.27 ± 1.41	
Type of onset^, prevalence (%):			
Supratentorial	333/1217 (27.36%)	27/86 (31.40%)	0.419
Optic pathway	321/1218 (26.35%)	18/86 (20.93%)	0.268
Brainstem	295/1217 (24.24%)	24/86 (27.91%)	0.445
Cerebellar	152/1217 (12.49%)	8/86 (9.30%)	0.384
Spinal cord	378/1217 (31.06%)	29/86 (33.72%)	0.607
Polysymptomatic	265/1219 (21.74%)	17/88 (19.32%)	0.594
Progression at onset^, prevalence (%)	199/1186 (16.78%)	21/84 (25.00%)	0.054
Brain MRI: number of T2w/FLAIR lesions^, prevalence (%)			
0 lesions	19/896 (2.12%)	0/62 (0.00%)	0.025
1–3 lesions	129/896 (14.40%)	7/62 (11.29%)	
4–10 lesions	349/896 (38.95%)	35/62 (56.45%)	
≥ 10 lesions	399/896 (44.53%)	20/62 (32.26%)	
Brain MRI: distribution of T2w/FLAIR lesions^, prevalence (%)			
Periventricular	735/812 (90.52%)	53/60 (88.33%)	0.580
Juxtacortical	534/792 (67.42%)	39/58 (67.24%)	0.977
Infratentorial	442/803 (55.04%)	31/58 (53.45%)	0.814
Corpus callosum	303/797 (38.02%)	21/57 (36.84%)	0.860
Brain MRI: atypical of lesions^, prevalence (%)	24/818 (2.93%)	0/66 (0.00%)	0.158
Brain MRI: number of T1w lesions^, prevalence (%)			
0 lesions	380/830 (45.78%)	27/59 (45.76%)	0.666
1–3 lesions	216/830 (26.02%)	19/59 (32.20%)	
4–10 lesions	167/830 (20.12%)	9/59 (15.25%)	
≥ 10 lesions	67/830 (8.07%)	4/59 (6.78%)	
Brain MRI: contrast lesions^, mean ± sd	0.81 ± 2.25	0.67 ± 1.15	0.599
Brain MRI: persistent contrast lesions^, prevalence (%)	41/896 (4.58%)	3/66 (4.55%)	0.991
Spine MRI: number of STIR lesions^, prevalence (%)			
0 lesions	300/842 (35.63%)	11/55 (20.00%)	0.006
1–3 lesions	437/842 (51.90%)	30/55 (54.55%)	
4–10 lesions	101/842 (12.00%)	13/55 (23.64%)	
≥ 10 lesions^§^	4/842 (0.48%)	1/55 (1.82%)	
Spine MRI: atypical of lesions^, prevalence (%)	8/440 (1.82%)	0/44 (0.00%)	0.367
Spine MRI: contrast lesions^, mean ± sd	0.24 ± 0.57	0.29 ± 0.65	0.467
MRI: Barkhof criteria^, prevalence (%)	781/973 (80.27%)	51/68 (75.00%)	0.295
Abnormal evoked potentials^, prevalence (%)			
VEPs	339/617 (54.94%)	23/45 (51.11%)	0.618
BAEPs	101/377 (26.79%)	5/16 (31.25%)	0.699
MEPs	109/317 (34.38%)	9/25 (36.00%)	0.879
SEPs	225/461 (48.81%)	12/28 (42.86%)	0.534
Positive OCBs^, prevalence (%)	639/782 (81.71%)	51/59 (86.44%)	0.362
Recruitment data			
Disease duration (years), mean ± sd	12.0 ± 9.0	8.9 ± 9.0	0.002
EDSS (score),			0.418
Median (range)	2.0 (0.0–9.5)	2.5 (0.0–8.0)	
Mean ± sd	2.64 ± 2.28	2.85 ± 2.23	
EDSS changes (point in score), median (range)	0.0 (− 3.5 to 6.5)	0.0 (− 4.0 to 6.0)	0.476
Time-gap from onset to EDSS, 4.0 (years), mean ± sd	7.1 ± 8.8	3.9 ± 4.4	0.049
Time-gap from onset to EDSS 6.0 (years), mean ± sd	9.5 ± 8.9	5.7 ± 5.2	0.132
Relapses in the first 3 years within onset, mean ± sd	1.90 ± 1.54	1.79 ± 1.46	0.575
Annual relapse rate, mean ± sd	0.72 ± 1.12	0.57 ± 0.60	0.353
Annual clinical visit rate, mean ± sd	2.27 ± 1.75	2.21 ± 1.52	0.785
Annual MRI scan rate, mean ± sd	0.99 ± 0.40	1.10 ± 0.44	0.020
Progression at follow-up, prevalence (%)	264/1218 (21.67%)	25/87 (28.74%)	0.125
Time on first DMT (years), mean ± sd	4.0 ± 4.7	3.1 ± 4.3	0.088
Number of DMTs, mean ± sd	1.91 ± 1.22	1.85 ± 1.19	0.662
Therapeutic approach, prevalence (%)			0.088
Induction	206/1090 (18.90%)	9/80 (11.25%)	
Escalation	884/1090 (81.10%)	71/80 (88.75%)	

^§^Adjacent categories with frequency < 5 were collapsed for p value calculation

At recruitment time, the disease duration was longer among WENA (p = 0.002), while OEG had higher EDSS score when an adjustment for disease duration and EDSS score at onset was applied (p = 0.044). WENA patients also reported a longer time-gap between onset and EDSS score of 4.0 (p = 0.013). Finally, OEG patients had undergone a higher number of annual MRI scans than WENA (p = 0.020).

Heterogeneity among MS Centres was statistically significant (variance = 2.21; 95% CI 0.44–11.14). The two-level variance component logistic regression model confirmed that OEG patients had a higher spine lesion load at onset (*1–3 lesions vs 0 lesions*: OR 3.30, p = 0.039, 95% CI 1.06–10.22) and a higher EDSS at last clinical follow-up (*4–6 vs 0–3.5*: OR 5.49; p = 0.033, 95% CI 1.15–26.24; > *6 vs 0–3.5*: OR 21.70; p = 0.005, 95% CI 2.58–182.75); while WENA patients reported a longer disease duration (> *10 vs* <  = *10 years*: OR 0.17; p = 0.018, 95% CI 0.04–0.74) (Table 3). We also noticed that, in OEG patients, a higher lesion load at onset correlated with a higher EDSS at last clinical follow-up (rho = − 0.122, p < 0.001).

**Table 3:** Comparison between White Europeans and North Americans (WENA) patients and other ethnical group (OEG) patients: adj ORs and 95% CIs

	Adj OR^§§^	95% CI	p value
Brain MRI: number of T2w/FLAIR lesions			
0–3 vs 4–10	1.32	0.35–4.87	0.679
0–3 vs ≥ 10	0.77	0.17–3.40	0.731
Spine MRI: number of STIR lesions			
0 vs 1–3 lesions	3.30	1.06–10.22	0.039
0 vs ≥ 4 lesions	2.53	0.49–12.90	0.265
Disease duration (years)			
≤ 10 vs > 10	0.17	0.04–0.74	0.018
EDSS (score) at follow-up			
0–3.5 vs 4–6	5.49	1.15–26.24	0.033
0–3.5 vs > 6	21.70	2.58–182.75	0.005
MRI Scan rate			
≤ 1 vs > 1	3.12	1.08–9.01	0.035

^§^“WENA” is the reference

As per native-Country economy, age at follow-up was higher in patients from HI Countries (p < 0.001), while we found higher prevalence of both psychiatric comorbidity and other CNS comorbidity in LMI group (p = 0.010; p = 0.013).

Clinical, paraclinical, and radiological characteristics of the groups at onset, diagnosis, and recruitment are detailed in Table 4. At onset, LMI patients had higher mean EDSS score as compared to the HI group, and higher proportion of progressive phenotype (p < 0.001). LMI also featured higher brain MRI activity at diagnosis, with 137/156 (87.8%) subjects with > 3 T2w lesions compared to 692/831 (83.3%) in the HI group (p = 0.008). Moreover, 13/163 (8.0%) LMI vs 31/820 (3.8%) HI patients had persistent contrast-enhancing lesions at diagnosis (p = 0.018).

**Table 4:** Comparison between patients born in high-income countries and patients born in middle-low-income countries

	High-income patients (n = 1109)	Middle-low income patients (n = 241)	p value
Demographic data			
Age, mean ± sd	45.6 ± 11.6	42.3 ± 11.7	< 0.001
Female:male (ratio)	804:305 (2.64)	169:72 (2.35)	0.457
Familiarity for AI disease, prevalence (%)	149/998 (14.93%)	40/202 (19.80%)	0.083
Comorbidity, prevalence (%)	526/1066 (49.34%)	104/194 (53.61%)	0.274
Psychiatric comorbidity, prevalence (%)	77/1069 (7.20%)	26/208 (12.50%)	0.010
Other CNS disease, prevalence (%)	27/1068 (2.53%)	12/208 (5.77%)	0.013
Onset and diagnosis data			
Age at onset, mean ± sd	30.2 ± 10.0	29.4 ± 10.3	0.279
Time-gap from onset to diagnosis gap (months), mean ± sd	34.6 ± 59.4	31.2 ± 60.7	0.438
EDSS (score)^			
Median (range)	2.0 (0.0–7.0)	2.0 (0.0–8.0)	< 0.001
Mean ± sd	1.99 ± 1.17	2.40 ± 1.43	
Type of onset^, prevalence (%)			
Supratentorial	292/1108 (26.35%)	74/231 (31.60%)	0.103
Optic pathway	284/1108 (25.63%)	62/232 (26.72%)	0.730
Brainstem	279/1108 (25.18%)	50/231 (21.65%)	0.256
Cerebellar	145/1108 (13.09%)	23/231 (9.96%)	0.191
Spinal cord	353/1108 (31.86%)	72/231 (31.17%)	0.838
Polysymptomatic	249/1108 (22.47%)	43/231 (18.30%)	0.159
Progression at onset^, prevalence (%)	166/1076 (15.43%)	58/225 (25.78%)	< 0.001
Brain MRI: number of T2w/FLAIR lesions^, prevalence (%)			
0 lesions	17/831 (2.05%)	2/156 (1.28%)	0.008
1–3 lesions	122/831 (14.68%)	17/156 (10.90%)	
4–10 lesions	317/831 (38.15%)	80/156 (51.28%)	
≥ 10 lesions	375/831 (45.13%)	57/156 (36.54%)	
Brain MRI: distribution of T2w/FLAIR lesions^, prevalence (%)			
Periventricular	653/720 (90.69%)	141/158 (89.24%)	0.574
Juxtacortical	473/702 (67.38%)	105/154 (68.18%)	0.847
Infratentorial	396/713 (55.54%)	81/154 (52.60%)	0.506
Corpus callosum	257/709 (36.25%)	68/151 (45.03%)	0.043
Brain MRI: atypical of lesions^, prevalence (%)	17/718 (2.37%)	7/179 (3.91%)	0.252
Brain MRI: number of T1w lesions^, prevalence (%)			
0 lesions	358/770 (46.49%)	57/145 (39.31%)	0.449
1–3 lesions	199/770 (25.84%)	44/145 (30.34%)	
4–10 lesions	148/770 (19.22%)	31/145 (21.38%)	
≥ 10 lesions	65/770 (8.44%)	13/145 (8.97%)	
Brain MRI: contrast lesions^, mean ± sd	0.79 ± 2.29	0.84 ± 1.54	0.734
Brain MRI: persistent contrast lesions^, prevalence (%)	31/820 (3.78%)	13/163 (7.98%)	0.018
Spine MRI: number of STIR lesions^, prevalence (%)			0.611
0 lesions	264/766 (34.46%)	55/159 (34.59%)	
1–3 lesions	403/766 (52.61%)	79/159 (46.69%)	
4–10 lesions	94/766 (12.27%)	24/159 (15.09%)	
≥ 10 lesions^§^	5/766 (0.65%)	1/159 (0.63%)	
Spine MRI: atypical of lesions^, prevalence (%)	5/386 (1.30%)	3/104 (2.88%)	0.256
Spine MRI: contrast lesions^, mean ± sd	0.24 ± 0.56	0.26 ± 0.64	0.603
MRI: Barkhof criteria^, prevalence (%)	701/885 (79.21%)	144/184 (78.26%)	0.774
Abnormal evoked potentials^, prevalence (%)			
VEPs	306/559 (54.74%)	63/112 (56.25%)	0.769
BAEPs	94/350 (28.86%)	12/47 (25.53%)	0.838
MEPs	98/295 (33.22%)	22/51 (43.14%)	0.175
SEPs	207/422 (49.05%)	35/73 (47.95%)	0.847
Positive OCBs^, prevalence (%)	581/712 (81.60%)	126/149 (84.56%)	0.391
RECRUITMENT DATA			
Disease duration (years)^, mean ± sd	12.0 ± 9.0	10.4 ± 8.7	0.016
EDSS (score)°			0.434
Median (range)	2.0 (0.0–9.5)	2.0 (0.0–8.0)	
Mean ± sd	2.63 ± 2.29	2.75 ± 2.17	
EDSS changes (point in score)°, median (range)	0.0 (− 3.5 to 6.5)	0.0 (− 4.0 to 6.0)	0.221
Time-gap from onset to EDSS 4.0 (years)°, mean ± sd	6.8 ± 7.8	6.1 ± 7.0	0.513
Time-gap from onset to EDSS 6.0 (years)°, mean ± sd	9.3 ± 8.9	8.0 ± 7.7	0.466
Relapses in the first 3 years within onset°, mean ± sd	1.96 ± 1.55	1.65 ± 1.43	0.007
Annual relapse rate°, mean ± sd	0.75 ± 1.19	0.54 ± 0.56	0.032
Annual clinical visit rate°, mean ± sd	2.29 ± 1.78	2.13 ± 1.39	0.214
Annual MRI scan rate°, mean ± sd	0.98 ± 0.39	1.08 ± 0.46	< 0.001
Progression at follow-up°, prevalence (%)	231/1103 (20.94%)	62/238 (26.05%)	0.084
Time on first DMT (years)°, mean ± sd	4.0 ± 4.8	3.5 ± 4.2	0.161
Number of DMTs°, mean ± sd	1.92 ± 1.24	1.80 ± 1.13	0.150
Therapeutic approach°, frequency (%)			0.074
Induction	194/987 (19.66%)	31/215 (14.42%)	
Escalation	793/987 (80.34%)	184/215 (85.58%)	

^§^Adjacent categories with frequency < 5 were collapsed for p value calculation

At recruitment, HI patients had a longer disease duration as compared to LMI (p = 0.016) and reported higher clinical activity as assessed by both the number of relapses within three years of disease onset (p = 0.007) and the ARR (p = 0.032). The mean number of annual MRI scans was higher in the LMI (p < 0.01).

Heterogeneity among centres was statistically significant (variance = 4.15; 95% CI 0.77–22.34). At the two-level variance component logistic regression model, only age at follow-up (> *45 vs* ≤ *45 years*: OR 0.27, p = 0.017, 95% CI 0.09–0.79) and the EDSS score at onset (> *5.0 vs 0.0–2.5*: OR 14.73, p = 0.032, 95% CI 1.27–171.02) statistically differed between the two groups after adjustment (Table 5).

**Table 5:** Comparison between patients from low-middle vs high income country: adj ORs and 95% CIs

	Adj OR^§§^	95% CI	p value
Age			
> 45 vs ≤ 45	0.27	0.19–4.60	0.017
Psychiatric_comorbidity			
Yes vs no	0.95	0.30–39.97	0.945
Other CNS comorbidity			
Yes vs no	3.49	0.44–3.81	0.315
EDSS (score) at onset			
0.0–2.5 vs 3.0–5.0	1.30	1.27–171.02	0.636
0.0–2.5 vs > 5.0	14.73	0.36–6.37	0.032
Progression at onset			
Yes vs no	1.52	0.36–6.37	0.570
Brain MRI: number of T2w/FLAIR lesions			
0–3 vs 4–10	1.39	0.39–4.99	0.614
0–3 vs ≥ 10	1.22	0.51–9.70	0.291
Brain MRI: distribution of T2w/FLAIR lesions	1.37	0.50–3.76	0.539
Corpus callosum			
Disease duration (years)			
≤ 10 vs > 10	0.97	0.35–2.66	0.948
EDSS (score) at follow-up			
0–3.5 vs 4–6	2.34	0.53–10.40	0.265
0–3.5 vs > 6	1.97	0.29–13.23	0.487
Relapses in the first 3 years within onset			
≤ 2 vs > 2	1.36	0.46–4.04	0.577
MRI Scan rate			
≤ 1 vs > 1	1.25	0.09–0.79	0.673

^§^“High income” is the reference

## Discussion

Migration studies focusing on the association between MS course and exposure to risk factors in both the Country of origin and in that of destination, have highlighted how among migrants, the disease clinical and radiological features tend to be intermediate between those of MS in their birthplace and in the Country of destination, and closer to the latter when migration occurs early in childhood [3, 16–19].

The International Organization of Migration (IOM) estimated that, in 2019, there were around 272 million international foreign-born patients in the World, who amounted to 3.5% of the global population, confirming an increasing trend registered since 1980 [20]. As a result, a rising number of foreign-born patients are referred to MS Centres worldwide. Nonetheless, in a recent systematic review by Onuorah et al., the authors reported that non-White people are constantly underrepresented in clinical trials, questioning whether this phenomenon could affect the generalisability of findings that are applied in clinical settings [13, 14].

Our study revealed that both OEG patients and patients born in LMI economies experience a more aggressive MS at disease onset. We found that OEG patients had a higher spinal cord MRI lesion load at onset. In line with previous studies reporting on the prognostic role of lesion load [21, 22], OEG patients had accumulated a more severe disability and in a shorter time-gap. Furthermore, these patients had undergone a higher number of annual MRI scans, also pointing to a more aggressive MS phenotype requiring a stricter monitoring of the disease activity [21, 23].

Patients who were born in a LMI Country had a higher disability at onset as compared to HI Country, but this difference disappeared at recruitment possibly depending on a similar clinical management of both groups across the Italian MS Centres, and independently from the Country of origin.

Evidence of an association between ethnicity and the geographic distribution of MS suggests that ethnicity may contribute to the risk for the development of MS [24]. The effect of ethnicity on the disease course is instead still controversial. African-American and Hispanic patients are shown to feature a worse prognosis than White patients, but these studies present important limitations, including referral centre bias and the lack of adjustment for socioeconomic status that can lead to overestimation of ethnical differences [25–27].

In our study, OEG MS subjects showed a more rapid clinical decline than the WENA group [21, 22]. An interaction between genetic and environmental factors likely plays a role in defining ethnic differences in health and disease, but the complex genetic-environmental susceptibility of immune-mediated/autoimmune diseases has not been fully elucidated yet. A more rapidly progressive course of immune-mediated diseases is reported in OEG patients [16, 25, 28]. Our results are in line with these findings, reporting that these patients had developed more severe disability over a shorter duration of the disease. However, OEG patients also had higher brain and spine MRI activity at disease onset. A strong association between MRI measures at baseline and clinical status at follow-up has been largely confirmed in studies conducted on the WENA populations [29–31]. In our study it was not possible to obtain data on white and grey matter volumes, but the number of lesions, a marker of disease activity and a predictor of disability accumulation, could be analysed [29–31]. In OEG patients the higher lesion load at baseline was associated with higher EDSS score at last clinical follow-up. Moreover, the retrospective collection of data does not allow to rule out that the registration of the date of onset was postponed due to a misdiagnosis among OEG. Indeed, diagnosing MS in OEG is still challenging considering the limitation of available data and their under-representation in clinical trial [32, 33]. Therefore, our findings may be confounded by a longer pre-clinical phase over which patients had developed more MS lesions and that could explain also the higher disability reported at the last clinical follow-up.

The comparison between LMI and HI Countries revealed that patients in the former group had a higher EDSS score at onset. Unfortunately, limited data are available on MS in LMI economies as most of the studies have been conducted in Western Europe and North America [4, 34]. In 2016, the Attendees at the International Workshop on Comorbidity in MS confirmed how the socio-economic status could account for relevant disparities in disability underlining that this status could accelerate brain aging and, potentially, disability progression in MS [35]. On the other hand and based on our findings, it is not possible to exclude that among the LMI the reported clinical onset is more frequently delayed than among HI and that therefore the time of diagnosis is also delayed. Information about the Country of diagnosis was available for only a small percentage of patients, and considering the low number of neurologists in LMI Countries, the first event could be misdiagnosed [4]. However, we did not find any differences in neurological disability at the last clinical follow-up. These findings suggest that patients of both groups had similar access to care and treatment opportunities across Italian MS Centres, independently of their birthplace, and that patients with a more aggressive disease at onset/diagnosis might have undergone a high-efficacy DMT. These data are in line with evidence from different HI Countries, supporting the belief that healthcare services and treatment strategies are equally available for MS patients who visit academic medical Centre or MS specialty Clinics [25, 36].

While the study offers valuable insights, it is important to acknowledge its limitations. One potential constraint is the presence of selection bias, as LMI foreign-born patients may also include undocumented foreign-born patients whose data might not be included in the analysis. In Italy, irregularly staying immigrants have access to essential level of healthcare system through a “foreign temporary present person” (*straniero temporaneamente presente*, STP) code. Nonetheless, the access to healthcare facilities for the management of chronic diseases by undocumented immigrants is often difficult to guarantee and the number of undocumented immigrants who access to the Italian National Health System remains low. This condition could underestimate the number of LMI patients included in our study.

A second limitation of the study was related to missing data for a few variables that should be responsible of biased results. To prevent this risk, we conducted a sensitivity analysis on multiple imputed data-sets. Complete-cases analysis was confirmed, so the reader can be confident about the unbiasedness of the study findings.

Overall, the results obtained through this Italian multicentre study suggest that the ethnic group, as well as the socio-economic status of the native Country could result in a different disease course. Nonetheless, the interpretation of data on foreign-born populations still remain difficult due to several factors, including the demographic and socio-economic characteristics of this population, the type of migration, and the lack or quality of available data [37]. In fact, migration results in pronounced changes in the migrants’ environmental risk factors, modifying their susceptibility to MS and the natural history of the disease. Moreover, OEG patients and patients born in LMI Country are under-represented in clinical trial and epidemiological studies and the available data regarding these population are still limited. Our results suggest that these variables should be considered in designing future clinical studies.

In conclusion, findings from this Italian multicentre study support, in line with mounting literature on the topic, that both ethnicity and native-Country economic status independently influence MS disease onset and course. Overall, our results favour the hypothesis that the socio-economic status and related cultural factors may change when patients migrate to a different Country and shape the disease evolution. Our findings ultimately suggest that moving from a LMI to HI Country improve the access to the healthcare facilities reducing the unbalance in disability outcome.

Migration studies are a valuable method not only to investigate environmental and genetic contributions in MS etiological research, but also the complexity of disease course and prognosis in migrant populations. In the era of personalised-medicine, a profound knowledge of factors associated to migration is a valuable instrument and an ethical approach to increase our capability to optimise the global management of MS. Indeed, a deeper knowledge of ethnical and socio-economic diversity would be essential to better design clinical trials and increase the overall generalisability of findings. To our knowledge, our study for the first time approaches this issues at individual—and not at population—level ultimately investigating the impact of exposures from Country of origin on a complex diseases, such as MS.

### Supplementary Information

Below is the link to the electronic supplementary material.

Supplementary file1 (DOCX 804 kb)

## Data Availability

Data reported in this study are anonymous for regulatory privacy reasons; they available upon reasonable request to the corresponding author.
