# Supplementary material for: The role of ethnicity and native-country income in multiple sclerosis: the Italian multicentre study (MS-MigIT)
Source: J Neurol. 2024 Feb 16;271(5):2182–94. doi: 10.1007/s00415-024-12214-6 (PMC11055772; doi:10.1007/s00415-024-12214-6)

**Supplemental Figure 1. The world maps report the native-country distribution of different ethnical groups included in the study population. Ethnicities were obtained from medical records as self-reported by patients at the first visit at MS Centre or obtained directly from patients.**


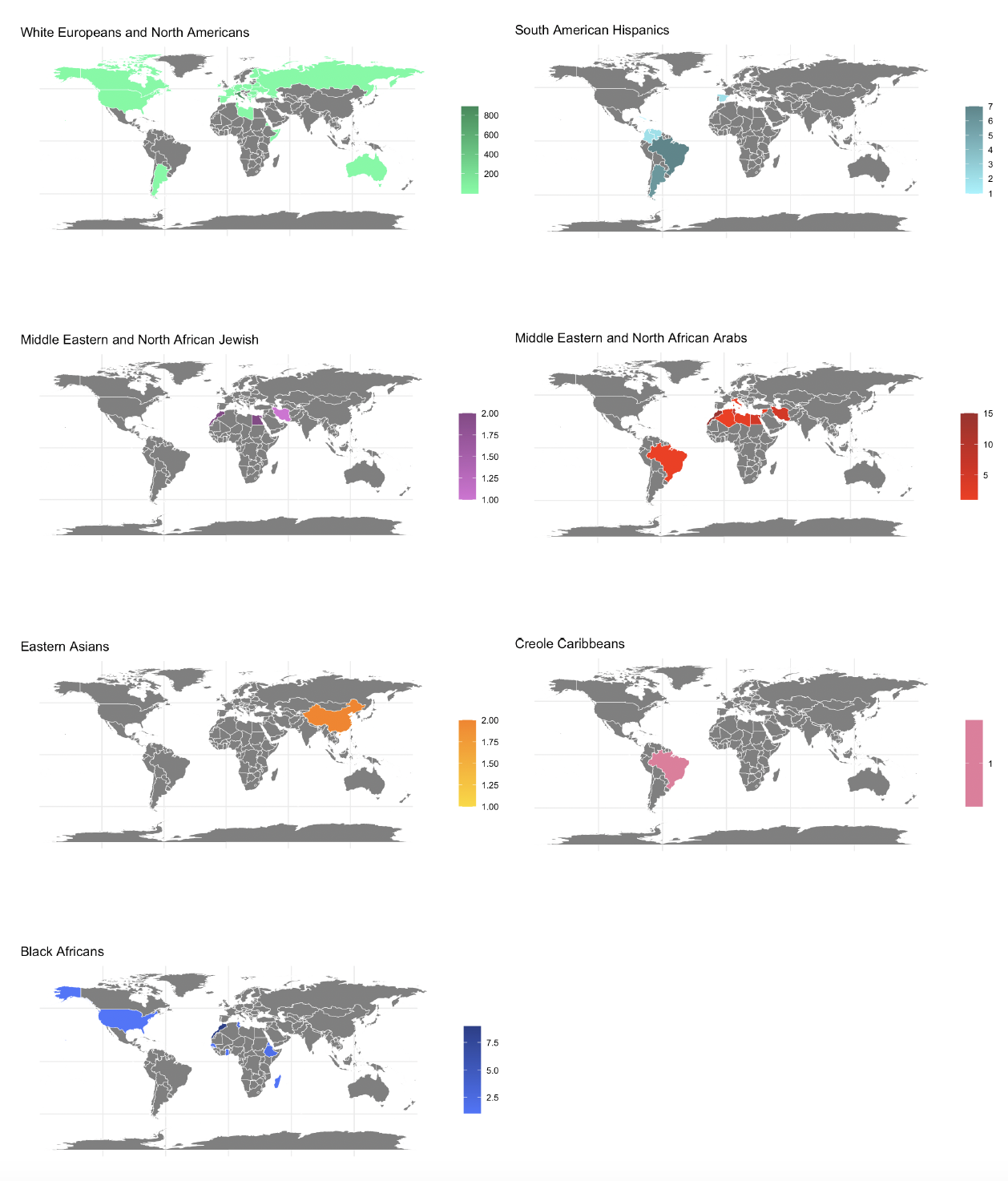


**Supplemental Figure 2. The world map reports the income of different countries included in the study population according to the 2018 World Bank Atlas. Low- and middle-income (LMI) economies are defined as those with a GNI per capita of less than United States (US) $12,056, while high-income (HI) economies are those with a GNI per capita of US $12,056 or higher.**


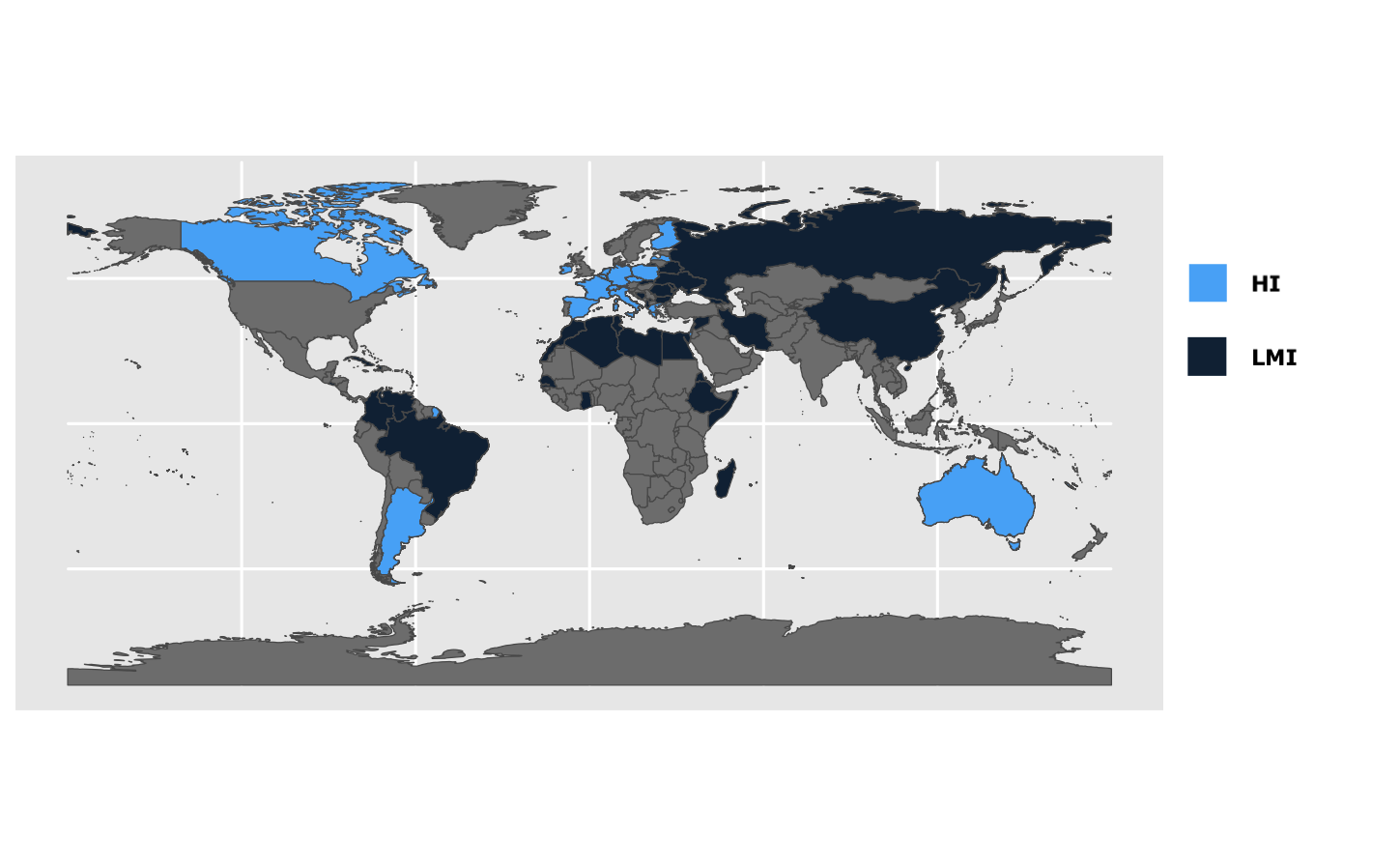

Supplement: Supplementary file 1 — Supplementary file1 (DOCX 804 kb) [file 415_2024_12214_MOESM1_ESM.docx]
